# Role of Imaging in the Diagnosis and Management of Complete Androgen Insensitivity Syndrome in Adults

**DOI:** 10.1155/2013/158484

**Published:** 2013-05-23

**Authors:** Marco Nezzo, Pieter De Visschere, Guy T'Sjoen, Steven Weyers, Geert Villeirs

**Affiliations:** ^1^Department of Radiology, University of Rome Tor Vergata, 00133 Rome, Italy; ^2^Department of Radiology, Ghent University Hospital, 9000 Ghent, Belgium; ^3^Department of Endocrinology, Ghent University Hospital, 9000 Ghent, Belgium; ^4^Department of Gynaecology, Ghent University Hospital, 9000 Ghent, Belgium

## Abstract

Complete androgen insensitivity syndrome is an X-linked recessive androgen receptor disorder characterized by a female phenotype with an XY karyotype. Individuals affected by this syndrome have normal female external genitalia but agenesis of the Müllerian duct derivatives, that is, absence of the Fallopian tubes, uterus, cervix, and the proximal part of the vagina, with presence of endoabdominal, labial, or inguinal testes. The estimated prevalence is between 1 and 5 in 100,000 genetic males. Complete androgen insensitivity syndrome can be diagnosed as a result of mismatch between the prenatal sex prediction and the phenotype at birth, can be detected by chance, or remain undetected until investigations for primary amenorrhea. Imaging can be important both to diagnose the pathology and to localize gonads prior to surgical treatment. In this paper, we present three cases of complete androgen insensitivity syndrome in adult women of 34, 22, and 38 years old.

## 1. Case 1

**Figure 1:**
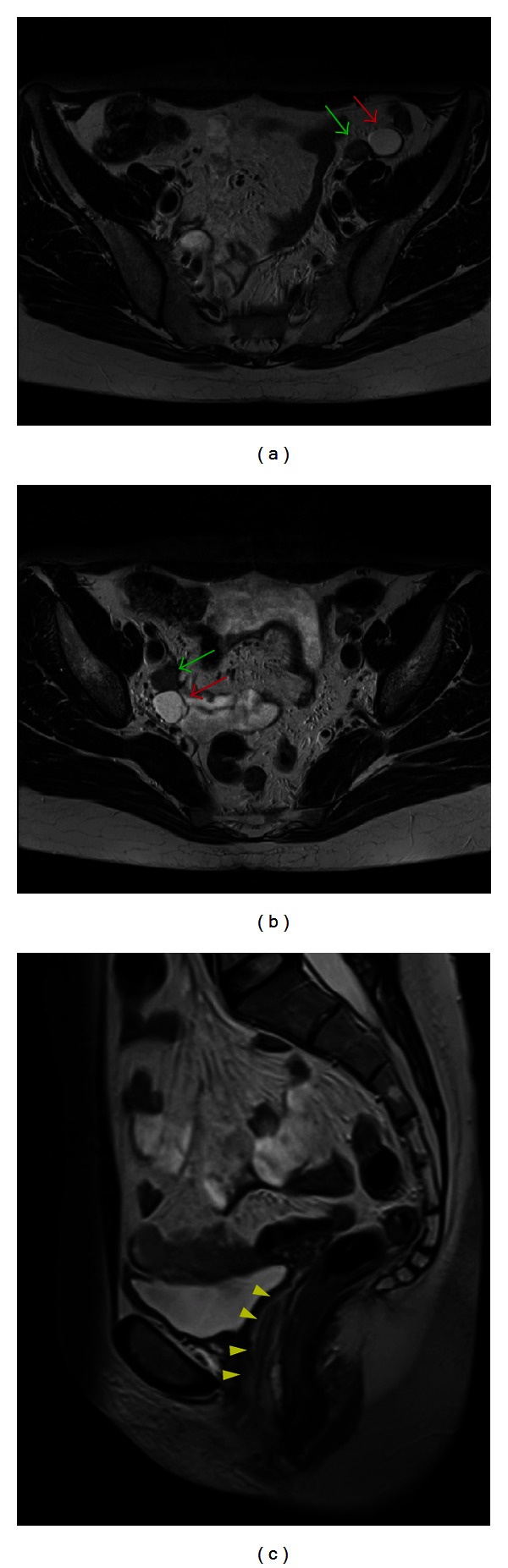
A 34-year-old female with CAIS syndrome. The axial T2-weighted images ((a) and (b)) show a left testis along the left external iliac artery and a right testis in the right obturator canal (green arrows) with adjoining cysts (red arrows). Sagittal image (c) shows absence of Müllerian structures and presence of the lower vagina (yellow arrowheads) (3T magnetic resonance imaging. Axial 4 mm fast T2-weighted images, TR/TE 5030/119 ms; sagittal 5 mm HASTE-sequence, TR/TE 6500/89).

A 34-year-old female was referred by the endocrinologist of our hospital. Her older sister had been diagnosed with complete androgen insensitivity syndrome (CAIS) at the age of 17. She had a female phenotype with normal external genitalia, normal breast development, and little axillary and pubic hair. The digital vaginal examination revealed a short, tight, and blind-ended vagina. An ultrasound (US) exam performed in another hospital had suggested the presence of gonads located in the pelvis. A magnetic resonance imaging (MRI) exam (Figure 1) was performed to plan a laparoscopic gonadectomy and showed two soft tissue structures suggestive for gonadal tissue located in the right obturator canal and along the left external iliac artery. A pericentimetric cyst was found adjacent to each gonad. Apart from the vagina, no development of Müllerian duct structures was observed.

## 2. Case 2

A 22-year-old female underwent two episodes of surgery for inguinal hernia before puberty. She was told that she has no uterus at the age of 15, but the diagnosis of CAIS was only recently made at another hospital. She had a male karyotype but normal female external genitalia and a vagina of normal size and depth. The patient had a normal height, weight, and breast development, with very subtle axillary and pubic hair.

**Figure 2:**
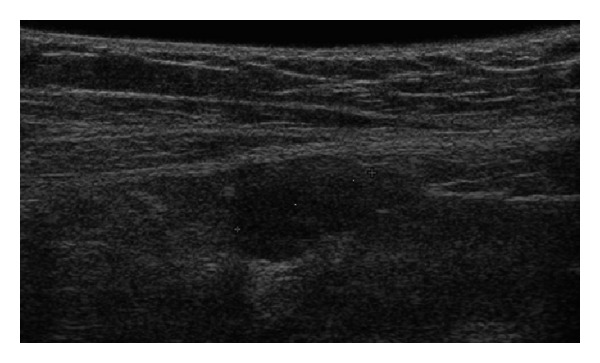
A 22-year-old girl/female with CAIS. Transabdominal ultrasonography (Hitachi EUB-8500, 14 MHz linear array probe) shows the presence of the right testis in the inguinal canal.

**Figure 3:**
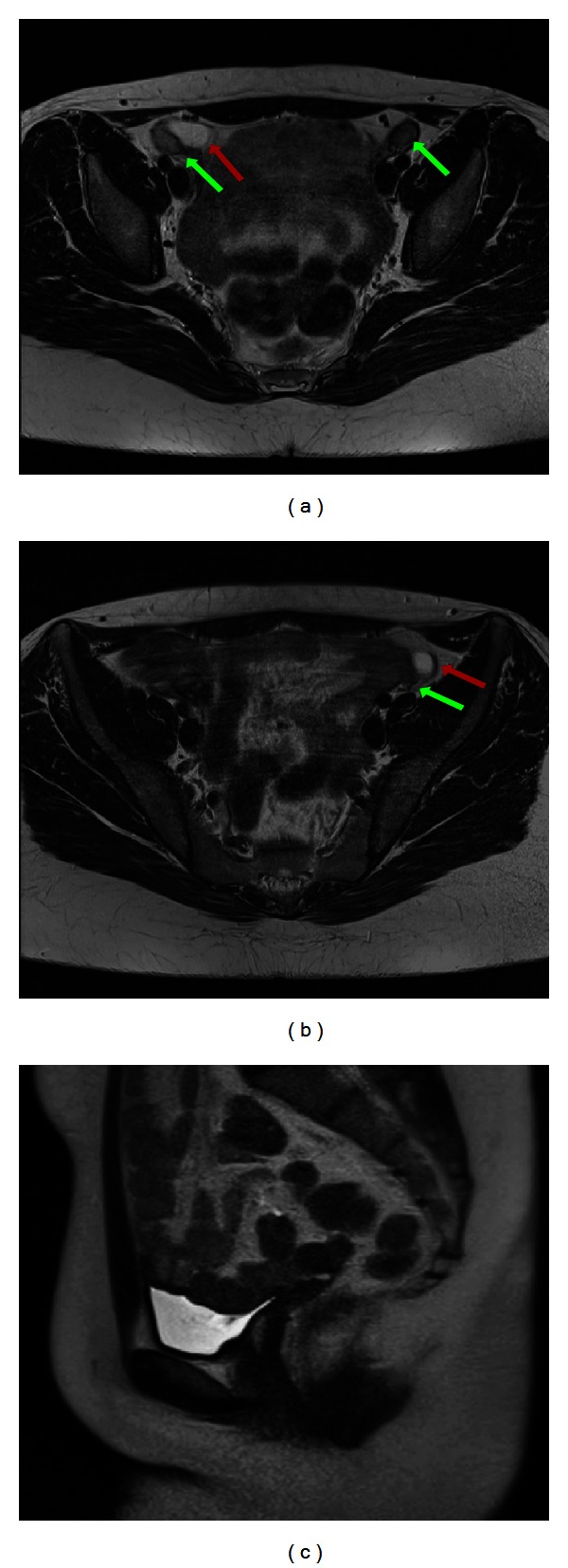
22-year-old girl/female affected by CAIS. Axial and coronal T2-weighted images ((a) and (b)) revealing the testes (green arrows) with adjoining cysts on both sides (red arrows). Sagittal image (c) showing absence of the Müllerian structures and the presence of the lower vagina (yellow arrowheads). (3T Magnetic Resonance Imaging. Axial 4 mm fast T2-weighted image, TR/TE 5030/119; sagittal 5 mm HASTE-sequence, TR/TE 6500/89).

She was now referred to our department by the gynecologist for an ultrasound and MRI exam prior to laparoscopic gonadectomy. US images (Figure 2) revealed a solid nodule suggestive of testicular tissue located in both inguinal regions. The MRI exam (Figure 3) confirmed a testicle in the deep inguinal ring on both sides and cysts adjacent to them. The uterus, cervix, and Fallopian tubes were absent, while the external genitalia and vagina had a normal appearance. Following gonadectomy, the patient was planned for hormonal substitution with estrogens.

## 3. Case 3

**Figure 4:**
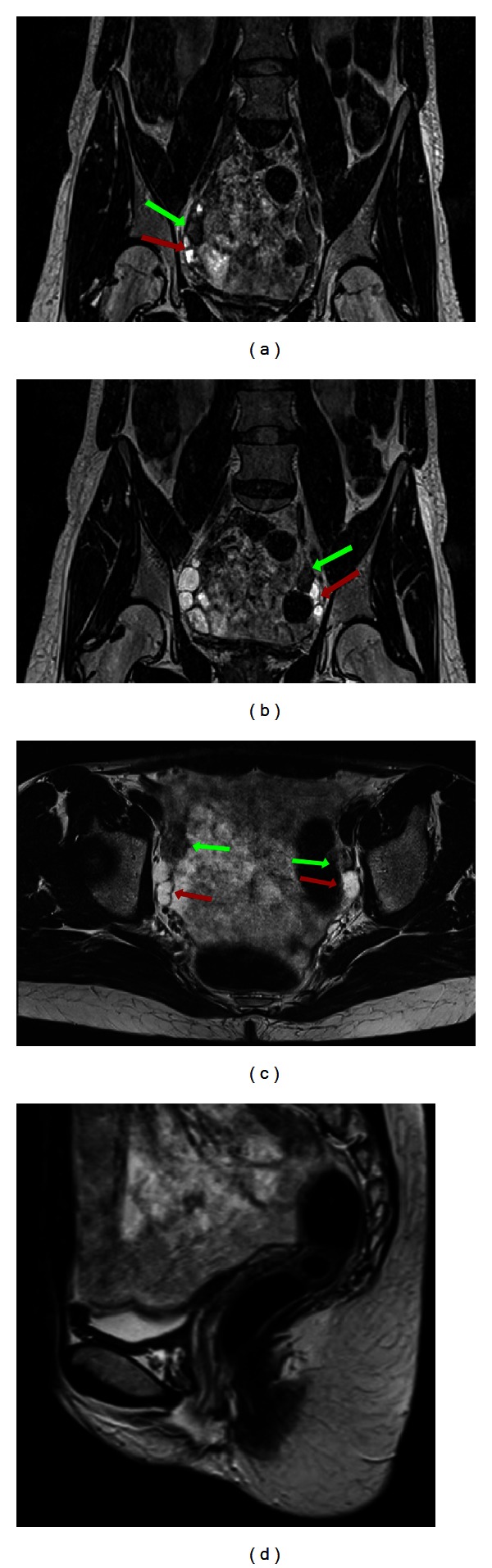
38-year-old girl/female affected by CAIS. MRI T2-weighted coronal ((a) and (b)) and axial (c) images demonstrating the presence of the testes (green arrows) and adjoining cysts (red arrows). Sagittal T2 weighted image (d) showing agenesis of the Müllerian structures and presence of the lower third of the vagina (yellow arrowheads). (3T Magnetic Resonance Imaging. Coronal 1 mm SPACE-sequence, TR/TE 1500/133; axial 4 mm fast-T2-weighted image, TR/TE 4500/119; sagittal 5 mm HASTE-sequence, TR/TE 6500/89).

A 38-year-old female was diagnosed with CAIS on the basis of primary amenorrhea. She presented with a male karyotype but female phenotype. She had normal external genitalia with a short vagina, normal height, weight, and breast development, no axillary hair, and very little pubic hair. She was referred to our department to exclude testicular malignancy, following her informed decision to undergo periodic MRI (watchful waiting) instead of preventive gonadectomy. The MRI exam (Figure 4) showed two testes with adjacent cystic components, each located in the obturator canal. There were no signs of malignancy. Müllerian derivatives were absent. A small Bartholin's cyst was noted on the left side of the perineum.

## 4. Discussion

Androgen insensitivity syndrome (AIS) is an X-linked recessive disorder caused by mutations of the androgen receptor (AR). The responsible gene has been localized at the proximal long arm of the X chromosome at Xq11-12 [1, 2]. According to the AR mutation database (http://www.mcgill.ca/androgendb), there are more than 1000 different mutations reported so far. The syndrome was first described in 1953 by Morris as testicular feminization, on the basis of his study of clinical features in 82 patients [3]. The syndrome was later given the name of androgen insensitivity syndrome.

Depending on the type of AR mutation, the failure of sexual differentiation can be either complete (CAIS), partial (PAIS), or mild (MAIS). We described 3 cases of CAIS, in which dysfunction of the AR is complete. CAIS patients have a male karyotype with female phenotype and despite testicular production of testosterone (T) and dihydrotestosterone (DHT), they do not develop either male genitalia or male secondary sexual characters; furthermore, since the testicular Sertoli cells produce Müllerian-inhibiting factor (MIF), they do not develop Müllerian structures. The testes can be located in the inguinal canal, sublabial, or intra-abdominal, and they are very commonly adjoined by small cystic pouches that are thought to be remnants of the Müllerian or Wolffian ducts [4]. The prevalence of CAIS is estimated between 1 and 5 in 100,000 genetic males on the basis of a proven molecular diagnosis [5] (Table 1).

Diagnosis of CAIS is obtained when a male karyotype is found in individuals with a female phenotype. They typically almost all have a female gender identity, normal breast development, little pubic and axillary hair, primary amenorrhea, and a blind-ending vagina. Although 77% of patients perceive their vagina as small and tight, only 35% actually have vaginal hypoplasia on genital examination [7], sometimes requiring vaginal dilatation or surgical reconstruction.

CAIS patients are usually taller than women without the syndrome but shorter than the male population [8]. They have a low bone mineral density before and/or after gonadectomy [9] that can be corrected by estrogen replacement therapy and/or calcium and vitamin D supplements. Their serum testosterone levels are within or above the normal range for males, and their luteinising hormone (LH) [10] and serum oestradiol concentrations are higher than in males but lower than in females without CAIS [11].

Karyotyping should be considered in all female infants diagnosed with a bilateral inguinal hernia [11, 12] because the latter is rare in normal female infants and associated with CAIS in 1-2% of cases during infancy [12, 13].

An intra-abdominal position of the testes is a well-known cause of malignant degeneration, but since the testes are normal and not dysgenetic in CAIS, prepubertal tumors are rare, with an incidence of 0.8% [14], whereas in adults the risk of gonadal malignancy increases and is estimated to be 14% (range 0% and 22%) [15].

Although it seems still prudent to perform the laparoscopic gonadectomy soon after puberty, the timing of gonadectomy is now becoming controversial in the literature and many women prefer to defer or decline the surgical procedure [15, 16]. In case of suspected malignant transformation before puberty, the testes can be removed and puberty induced with estrogen replacement.

CAIS differs from PAIS because of the presence of a hypospadic micropenis and a bifid scrotum that may contain the testes. MAIS is not associated with male genital anomalies but presents as infertility [17].

A very common differential diagnosis of CAIS is the Mayer-Rokitansky-Kuster-Hauser (MRKH) syndrome. Patients affected by the MRKH syndrome have a female karyotype and normal female external genitalia and ovaries (located high along the pelvic side wall), but with congenital aplasia of the uterus and the upper part of the vagina (Table 2) [6].

Transabdominal US can be used as a first-line examination for CAIS to assess the absence of Müllerian structures and to locate the testes. Since US is operator dependent and can remain inconclusive [18], MRI is the study of choice, with a reported accuracy up to 100% for the evaluation of Müllerian duct anomalies [19]. MRI provides detailed anatomic information due to its superior tissue characterization and multiplanar capability. US and MRI have an equal sensitivity for depicting pelvic gonads, but MRI has higher sensitivity for the localization of intra-abdominal gonads [20]. Sagittal and transverse T2-weighted images (T2WI) and transverse T1-weighted images (T1WI) are used to reveal the absence of the uterus, to evaluate the vagina, to assess the presence and location of the testes (which usually are hypointense on T1WI and slightly hyperintense on T2WI), and to exclude testicular malignancy [21] (Table 1). Furthermore, MRI is useful to plan a gonadectomy and, in addition to tumour markers, for a watchful waiting in patients who refuse surgical removal of the testes.

## 5. Teaching Point

CAIS is a disorder characterized by a female phenotype with an XY karyotype. Its diagnosis and management are typically established in a multidisciplinary team specialised in disorders of sexual differentiation. US can be used as a first-line evaluation of the Müllerian structures. However, MRI is considered as the gold standard because of its higher accuracy in the evaluation of Müllerian abnormalities. It plays a key role in the planning of laparoscopic gonadectomy by revealing the presence and location of the testes and in watchful waiting in patients who refuse gonadectomy.

## Figures and Tables

**Table 1:** Summary table for complete androgen insensitivity syndrome (CAIS).

Etiology	X-linked recessive androgen receptor dysfunction
Incidence	1/20,400 to 1/99,100
Gender ratio	Genetic males
Age predilection	Congenital syndrome
Risk factors	Unknown
Treatment	Testicular removal after puberty and estrogen replacement. Vaginal dilatation or surgical reconstruction. Psychological support often needed. Alternative: watchful waiting
Prognosis	Good if testes are timely removed
Imaging findings	Transabdominal US Absence of Mullerian structures Presence of testes (usually smaller and slightly hypoechoic relative to normal testes) MRI *Axial plane*: absence of Mullerian structures, presence of testes and female external genitalia Evaluation of incidental testicular malignancy *Sagittal plane*: absence of uterus. Evaluation of presence and length of the lower vagina *Coronal plane*: presence of testes and female external genitalia. Absence of Mullerian structures

**Table 2:** Differential diagnosis for complete androgen insensitivity syndrome (CAIS). Adapted from Gottlieb et al. [6].

Type	External genitalia “synonyms”	Findings
CAIS	Female (“testicular feminization”)	XY karyotype Absence of Mullerian structures Abdominal or inguinal testes Female external genitalia Breast development Blind-ended vagina Scant or absent pubic and/or axillary hair

	Predominantly female (“incomplete AIS”)	XY karyotype Absence of Mullerian structures Breast development Clitoromegaly and labial fusion Distinct urethral and vaginal openings or a urogenital sinus
PAIS	Ambiguous	XY karyotype Absence of Mullerian structures Microphallus (<1 cm) with clitoris-like underdeveloped glans; labia majora-like bifid scrotum Descended or undescended testes Perineoscrotal hypospadias or urogenital sinus Gynecomastia/development of breast
	Predominantly male	XY karyotype Absence of Mullerian structures Simple (glandular or penile) or severe (perineal) “isolated” hypospadias with a normal-sized penis and descended testes or severe hypospadias with micropenis, bifid scrotum, and either descended or undescended testes Gynecomastia in puberty

MAIS	Male (“undervirilized male syndrome”)	XY karyotype Absence of Mullerian structures Impaired spermatogenesis and/or impaired pubertal virilization Gynecomastia in puberty

MRKH	Female	XX karyotype Absence of uterus and upper vagina Presence of female external genitalia and ovaries Normal breast development

## Abbreviations

AIS: Androgen insensitivity syndrome
CAIS: Complete androgen insensitivity syndrome
US: Ultrasound
MRI: Magnetic resonance imaging
AR: Androgen receptor
PAIS: Partial androgen insensitivity syndrome
MAIS: Mild androgen insensitivity syndrome
T: Testosterone
DHT: Dihydrotestosterone
MIF: Müllerian-inhibiting factor
LH: Luteinising hormone
MRKH: Mayer-Rokitansky-Kuster-Hauser
3*β*HSD: 3-Beta-hydroxysteroid dehydrogenase deficiency
WI: Weighted imaging.
